# Effects of different doses of erythropoietin in patients with myelodysplastic syndromes: A propensity score‐matched analysis

**DOI:** 10.1002/cam4.2638

**Published:** 2019-10-27

**Authors:** Enrico Balleari, Rosa Angela Filiberti, Chiara Salvetti, Bernardino Allione, Emanuele Angelucci, Marco Bruzzone, Tullio Calzamiglia, Marina Cavaliere, Maurizio Cavalleri, Daniela Cilloni, Marino Clavio, Elena Crisà, Anna Da Col, Paolo Danise, Federica Pilo, Dario Ferrero, Carlo Finelli, Daniela Gioia, Roberto Massimo Lemoli, Elisa Masiera, Emanuela Messa, Maurizio Miglino, Pellegrino Musto, Esther Natalie Oliva, Antonella Poloni, Flavia Salvi, Alessandro Sanna, Marco Scudeletti, Rodolfo Tassara, Valeria Santini

**Affiliations:** ^1^ Fondazione Italiana Sindromi Mielodisplastiche (FISM) Bologna Italy; ^2^ UO Internal Medicine Ospedale Policlinico San Martino‐IRCCS Genova Italy; ^3^ UO Clinical Epidemiology Ospedale Policlinico San Martino‐IRCCS Genova Italy; ^4^ UO Hematology University of Turin Torino Italy; ^5^ UO Hematology Molinette Hospital Torino Italy; ^6^ UO Hematology Ospedale Policlinico San Martino‐IRCCS Genova Italy; ^7^ UO Internal Medicine—ASL 1 Sanremo (IM) Italy; ^8^ UO Internal Medicine—ASL 2 Savona Italy; ^9^ UO Internal Medicine—ASL 4 Sestri Levante (GE) Italy; ^10^ UO Department of Clinical and Biological Sciences University of Turin Torino Italy; ^11^ UO Clinic of Hematology Department of Internal Medicine University of Genoa Ospedale Policlinico San Martino‐IRCCS Genova Italy; ^12^ UO Hematology Nocera Hospital Nocera Inferiore Italy; ^13^ UO Hematology—P.O. Oncologico Businco AOG. Brotzu Cagliari Italy; ^14^ UO Hematology AOU Policlinico Sant'Orsola‐Malpighi University of Bologna Bologna Italy; ^15^ UO Internal Medicine ASLTo4 Carmagnola Italy; ^16^ Regional Department of Hematology IRCCS‐CROB Referral Cancer Center of Basilicata Rionero in Vulture (Pz) Italy; ^17^ UO Hematology Grande Ospedale Metropolitano “Bianchi Melacrino Morelli” Reggio Calabria Italy; ^18^ UO Hematology Università Politecnica delle Marche Ancona Italy; ^19^ UO Hematology SS. Antonio e Biagio Hospital Alessandria Italy; ^20^ Ematologia Ospedale di Livorno Livorno Italy; ^21^ MDS Unit AOU Careggi University of Florence Firenze Italy

**Keywords:** anemia, erythropoietin, myelodysplastic syndromes

## Abstract

**Background:**

Erythropoiesis‐stimulating agents effectively improve the hemoglobin levels in a fraction of anemic patients with myelodysplastic syndromes (MDS). Higher doses (HD) of recombinant human erythropoietin (rhEPO) have been proposed to overcome suboptimal response rates observed in MDS patients treated with lower “standard doses” (SD) of rhEPO. However, a direct comparison between the different doses of rhEPO is lacking.

**Methods:**

A cohort of 104 MDS patients treated with HD was retrospectively compared to 208 patients treated with SD in a propensity score‐matched analysis to evaluate hematological improvement‐erythroid (HI‐E) rate induced by the different doses of rhEPO. The impact of rhEPO doses on survival and progression to leukemia was also investigated.

**Results:**

Overall HI‐E rate was 52.6%. No difference was observed between different rhEPO doses (*P* = .28) in matched cohorts; in a subgroup analysis, transfusion‐dependent patients and patients with higher IPSS‐R score obtained a higher HI‐E rate with HD, although without significant impact on overall survival (OS). Achievement of HI‐E resulted in superior OS. At univariate analysis, a higher HI‐E rate was observed in transfusion‐independent patients (*P* < .001), with a lower IPSS‐R score (*P* < .001) and lower serum EPO levels (*P* = .027). Multivariate analysis confirmed that rhEPO doses were not significantly related to HI‐E (*P* = .26). There was no significant difference in OS or progression to leukemia in patients treated with HD vs SD.

**Conclusion:**

SD are substantially equally effective to HD to improve anemia and influencing survival in MDS patients stratified according to similar propensity to be exposed to rhEPO treatment.

## Graphical abstract

When compared in a propensity‐score matched analysis, standard doses of rhEPO showed similar effects to those of higher doses in anemic MDS patients. Achievement of favorable response with hematologic improvement in this clinical scenario is possible by reducing drug doses and costs.

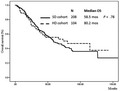

## 1 INTRODUCTION

Anemia is the major clinical concern for patients with myelodysplastic syndromes (MDS). It is present in about two of three patients at diagnosis, eventually rendering most of them transfusion dependent.^1^ Moreover, anemia is the main cause of both morbidity and mortality^2^ in MDS patients with a lower risk of progression according to the International Prognostic Scoring System (IPSS)^3^ and to its revised form (IPSS‐R).^4^

Erythropoietic‐stimulating agents (ESAs), in particular recombinant human erythropoietin (rhEPO), have been used to overcome anemia in MDS patients since the last three decades, soon after it became available in clinics for the treatment of anemia due to renal failure.^5^ ESAs have been shown to improve the clinical outcome of anemic MDS patients, ameliorating their quality of life,^6^, ^7^ and possibly exerting a positive impact on survival in patients achieving a significant increase in hemoglobin (Hb) and/or a reduction of transfusion need.^8^, ^9^ In 2018 rhEPO has been approved by EMA for IPSS lower‐risk MDS with endogenous levels <200 U/L and Hb <10 g/dL.

Unfortunately, not all patients treated with ESAs respond. Early studies explored the effects of weight‐adjusted doses of rhEPO in MDS anemic patients. Using these doses, usually slightly inferior to 30‐40.000 IU weekly, an erythroid response was obtained in 15%‐25% of MDS patients.^10^ Although encouraging, these results were inferior to more recent ones reporting a response rate >50%.^11^, ^12^ Higher response rate to ESAs currently observed is due to the better selection of MDS patients based on criteria developed and consolidated over the years.^9^ The use of doses of rhEPO higher than 40.000 IU is also deemed responsible for the improvement of response rates,^13^, ^14^, ^15^ and it has been recently recommended.^16^ The real clinical impact of higher vs lower doses of ESAs has never been evaluated in a randomized study or in a pair‐matched comparison, and we hypothesized that this type of analysis would clarify the role of different ESA doses in influencing response, overall survival, and progression to AML.

We thus retrospectively assessed the impact of higher vs lower doses of rhEPO on response to therapy and survival in two groups of MDS patients matched for clinical characteristics determining propensity to receive ESAs treatment. We limited this evaluation to rhEPO, given the scarce use of darbepoetin for MDS patients in Italy.

## 2 PATIENTS AND METHODS

### 2.1 Patient selection and data collection

This retrospective propensity‐matched cohort study was conducted using the Italian nation‐wide dataset of Fondazione Italiana Sindromi Mielodisplastiche (FISM‐Onlus), which includes more than 5000 MDS patients enrolled in the Italian Network of regional MDS registries (https://ClinicalTrials.gov Identifier: NCT02808858). The study was approved by the Ethics Committee and conducted according to national regulations for retrospective studies.

A cohort of MDS anemic patients treated with higher doses (HD) of rhEPO, defined as 40.000 IU twice a week, within 6 months from diagnosis was first identified. A second cohort, with a similar propensity to be treated with rhEPO according to clinical parameters known to influence the choice for a trial with ESAs^17^, ^18^ and treated with lower, “standard doses” (SD), defined as 40.000 IU weekly, was compared with the first one by using a 2:1 propensity score matching. Hematological improvement‐erythroid (HI‐E) was evaluated after 3 months of therapy, applying IWG 2006 criteria.^19^

### 2.2 Statistical analysis

Demographics and clinical characteristic of the patients were summarized as median (range) for continuous variables and number (%) for categorical variables. Relationships between categorical variables were examined by means of chi‐square test. Univariate analyses were performed to evaluate the response to therapy, progression to acute myeloid leukemia (AML), and prognostic impact on survival of individual and clinical variables. Overall survival (OS) was estimated using as survival time the difference (in months) between the date of death or last follow‐up and the date of start of therapy. Patients lost to follow‐up were censored at the date of their last visit. Kaplan‐Meier method was applied in univariate analysis to estimate survival probabilities and log‐rank test was carried out to assess heterogeneity within each prognostic factor. The cumulative 1‐, 2‐, and 3‐year survival probabilities were estimated.

To overcome mis‐estimating response to therapy and OS due to possible differences in patient baseline parameters between the two groups, a propensity score for each patient was calculated by a multivariable logistic regression analysis after allowance for age, MDS WHO 2008 classification, bone marrow blasts (<5% vs ≥5%), endogenous EPO (>200 vs ≤200 mU/mL), transfusion dependency (yes vs no), Hb (>8 vs ≤8 g/dL), ferritin (>350 vs ≤350 µg/L), and IPSS score (intermediate 1 or higher vs low). These variables were selected as relevant and conditioning predictors of rhEPO dose choice. The logistic regression coefficients of the variables that were significant in the model were then used to compute the propensity score.^20^ This score represents the probability that a patient would receive treatment with higher rhEPO doses based on variables which were suspected to influence group assignment. A 1:2 matched study group was created with the use of the nearest approach.

To estimate the effect of treatment dose on HI‐E and progression to AML in matched‐pair analysis, a multivariate analysis was performed by the random intercept logistic regression modeling for clustered data, adjusting for sex. The associated results are reported as odds ratio (OR) with 95% confidence intervals (CI). The effect of treatment doses and of sex on overall survival was estimated through the random intercept Cox regression modeling and the results are reported as hazard ratios (HR) with 95% CI.

A two‐tailed *P*‐value <.05 was considered statistically significant. All analyses were performed using SPSS software v. 20 by IBM.

## 3 RESULTS

### 3.1 Characteristics of the study group of MDS patients

At the time of data lockup (31 December 2016), 445 anemic patients (104 treated with HD and 341 with SD) with complete clinical annotations in the FISM registry satisfied the eligibility criteria and entered the study. Baseline patient characteristics, before matching, are reported in Table 1.

**Table 1:** Clinical characteristics of the patients at diagnosis. All patients (n = 445)

	Therapy		*P*
	Standard dose N (%)	High dose N (%)	
Total	341 (76.6)	104 (23.4)	
Sex			<.001
Male	179 (52.5)	77 (74.0)	
Female	162 (47.5)	27 (26.0)	
Age median (range)	75 (39‐98)	75 (30‐96)	
≤75	176 (51.6)	57 (54.8)	.58
>75	165 (48.4)	47 (45.2)	
WHO classification			
RA	132 (38.7)	30 (28.8)	
RARS	38 (11.1)	17 (16.5)	
RCMD	102 (29.9)	32 (31.1)	
RAEB1	33 (9.7)	15 (14.6)	
RAEB2	12 (3.5)	3 (2.9)	
MDS with isolated 5q‐	20 (5.9)	4 (3.9)	
MDS‐U	4 (1.2)	2 (1.9)	
Not available	—	1 (1.0)	
Hemoglobin median (range)g/dL	9.1 (2‐12.8)	8.9 (5.1‐13.1)	
≤8	74(21.7)	30(28.8)	.15
>8	267(78.3)	74 (71.2)	
<9	203 (59.5)	64 (61.5)	.73
>9	138 (40.5)	40 (38.5)	
<10	304 (89.1)	97 (93.3)	.26
>10	37 (10.9)	7 (6.7)	
Bone marrow blasts (%)			.55
<5	287 (84.2)	85 (81.7)	
≥5	54 (15.8)	19 (18.3)	
Transfusion dependency			.60
No	259 (76.0)	76 (73.1)	
Yes	82 (24.0)	28 (26.9)	
IPSS score risk			.013
Low	205 (60.1)	46 (44.2)	
Intermediate 1	112 (32.8)	52 (50.0)	
Intermediate 2	22 (6.5)	6 (5.8)	
High	2 (0.6)	—	
IPSS‐R score risk			.26
Very low	74 (21.7)	22 (21.2)	
Low	162 (47.5)	39 (37.5)	
Intermediate	68 (19.9)	30 (28.8)	
High	27 (7.9)	8 (7.7)	
Very high	10 (2.9)	5 (4.8)	
Ferritin median (range) μg/L	288 (4‐4985)	321.5 (6‐1600)	
≤350	200 (58.7)	47 (45.2)	.018
>350	141 (41.3)	57 (54.8)	
EPO median (range) mU/mL	59.0 (2‐3420)	75 (1‐1700)	
≤200	289 (84.8)	80 (76.9)	.074
>200	52 (15.2)	24 (23.1)	

### 3.2 Outcomes of the propensity score‐matched cohorts

All the 104 MDS patients treated with HD were matched with 208 patients treated with SD in a 1:2 fashion. Detailed characteristics of these two cohorts at the time of starting treatment are shown in Table 2. After matching, all covariates were well balanced, with nonsignificant difference in therapy‐related variables between the two cohorts.

**Table 2:** Clinical characteristics of patients after propensity score matching (n = 312) at the start of rhEPO treatment

	Propensity score‐matched patients		
	Therapy		*P*
	Standard dose N (%)	High dose N (%)	
Total	208 (66.7)	104 (33.3)	
Sex			<.001
Male	106 (51.0)	77 (74.0)	
Female	102 (49.0)	27 (26.0)	
Age median (range)			1.0
<75	114 (55.1)	57 (54.8)	
>75	93 (44.9)	47 (45.2)	
WHO classification			.90
RA	75 (36.1)	30 (28.8)	
RARS	26 (12.5)	17 (16.5)	
RCMD	60 (28.8)	32 (31.1)	
RAEB1	27 (13.0)	15 (14.6)	
RAEB2	5 (2.4)	3 (2.9)	
MDS with isolated 5q‐	11 (5.3)	4 (3.9)	
MDS‐U	4 (1.9)	2 (1.9)	
Not available	—	1 (1.0)	
Hemoglobin median (range) g/dL			
≤8	47(22.6)	30(28.8)	.26
>8	161(77.4)	74(71.2)	
<9	127 (61.1)	64 (61.5)	1.0
>9	81 (38.9)	40 (38.5)	
<10	188 (90.4)	97 (93.3)	.52
>10	20 (9.6)	7 (6.7)	
Bone marrow blasts (%)			1.0
<5	169 (81.3)	85 (81.7)	
>5	39 (18.8)	19 (18.3)	
Transfusion dependency			.89
No	149(71.6)	76 (73.1)	
Yes	59 (28.4)	28 (26.9)	
IPSS score risk			.44
Low	104 (50.0)	46 (44.2)	
Intermediate 1	92 (44.2)	52 (50.0)	
Intermediate 2	10 (4.8)	6 (5.8)	
High	2 (1.0)	—	
IPSS‐R score risk			.72
Very low	41 (19.7)	22 (21.2)	
Low	91 (43.8)	39 (37.5)	
Intermediate	51 (24.5)	30 (28.8)	
High	19 (9.1)	8 (7.7)	
Very high	6 (2.9)	5 (4.8)	
Ferritin median (range) μg/L			1.0
≤350	95 (45.7)	47 (45.2)	
>350	113 (54.3)	57 (54.8)	
EPO median (range) mU/mL			.67
≤200	165 (79.3)	80 (76.9)	
>200	43 (20.7)	24 (23.1)	

### 3.3 Achievement of HI‐E according to ESA doses

Overall, HI‐E was observed in 164 matched patients (52.6%). Nonsignificant differences were observed with regard to rhEPO doses: HI‐E was obtained in 54.8% of patients with SD vs 48.1% with HD, respectively (*P* = .28).

At univariate analysis, a statistically significant higher HI‐E was observed in patients with transfusion independence (no vs yes, *P* < .001), with an IPSS‐R lower‐risk score category (very low‐low vs intermediate‐very high, *P* < .001), and with lower serum EPO concentration (≤200 vs >200, *P* = .027). A trend to higher HI‐E was found in patients with <5% of marrow blasts (*P* = .08). Fewer responses were observed in patients with del (5q) and RAEB2 (20% and 25%, respectively) which were significantly different to response rates observed in RA (with or without ringed sideroblasts, RARS) and RCMD cases (58. 8% and 47.6%, respectively, *P* = .04) (Table S1). Multivariate analysis taking into consideration rhEPO doses, transfusion dependency, serum EPO levels, marrow blast percentage, WHO classification, and IPSS‐R, confirmed the predictive value of transfusion dependency (no vs yes: OR = 1.71, 95% CI 1.30‐2.25; *P* < .001) and IPSS‐R (very low‐low vs higher risk: OR = 1.45, 95% CI 1.03‐2.06, *P* = .035) while patients with del (5q) were confirmed to have a lower response rate (OR: 0.23, 95% CI 0.07‐0.79, *P* = .020). rhEPO doses were not significantly correlated with HI‐E (*P* = .39) (Table S2).

### 3.4 Overall survival according to ESA doses and response

**Figure 1:**
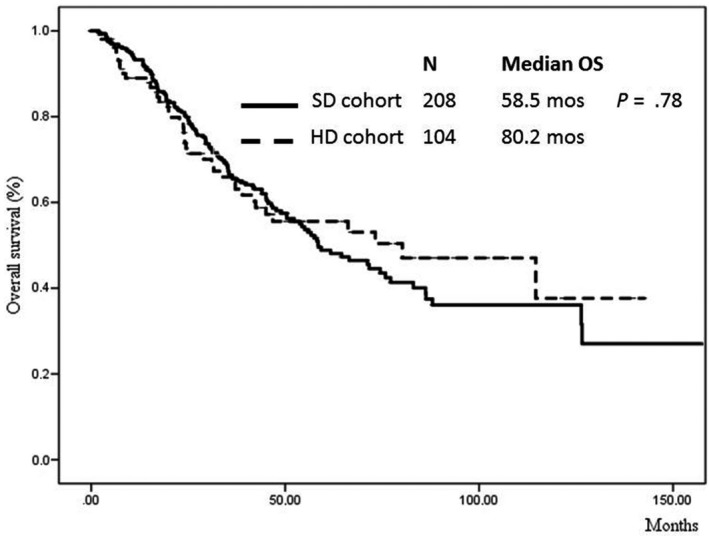
Overall survival of propensity score‐matched patients according to rhEPO doses

Median OS was 64.6 months (95% CI 49.2‐79.9 months). After a median observation time of 44.2 months (range 1.6‐156.8) and 26.7 months (range 1.8‐114.6) for censored and deceased patients, respectively, 133 patients (42.6%: 44.2% in SD and 39.4% in HD cohorts, respectively) died. One‐, 2‐ and 3‐year survival probabilities were 80%, 66%, and 37%, respectively. A no significant trend to longer OS was observed for the HD vs SD cohort (80.2 months, 95% CI 31.1‐129.3 for HD vs 58.5 months, 95% CI 42.3‐74.7 for SD; *P* = .78; Figure 1).

**Figure 2:**
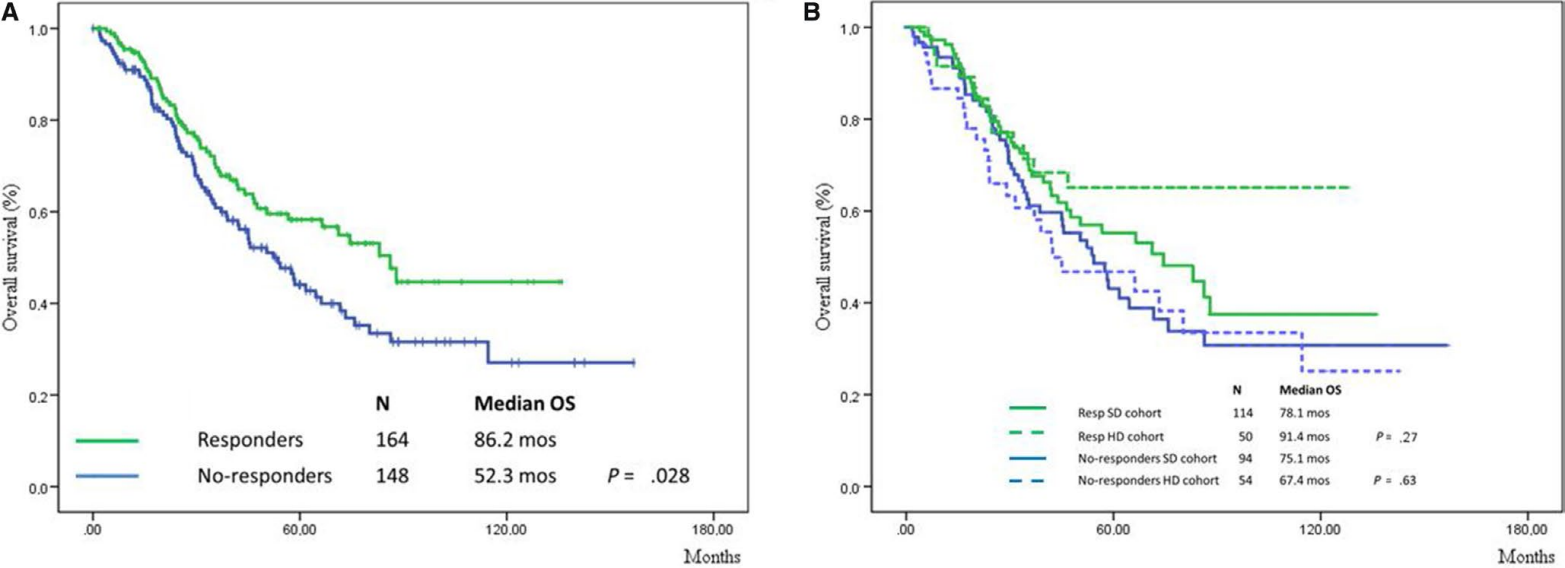
Overall survival of patients according to erythroid response in both cohorts (A) and within different subgroups according to different rhEPO doses (B)

At univariate analysis, female gender (*P* = .010), younger age (≤75 year, *P* < .001), low ferritin levels (≤350, *P* = .010), transfusion independence (*P* < .001), lower marrow blast percentage (≤5%, *P* = .001), and IPSS‐R lower‐risk category (low‐very low vs intermediate‐very high, *P* < .001) were all significant positive prognostic factors for OS. Median OS was significantly shorter for RAEB and RCMD cases (31 and 40 months, respectively) compared to RA and RARS cases (83 and 95 months, respectively, *P* = .002) (Table S3). Patients achieving HI‐E had a longer OS (median OS: 86.2 vs 52.3 m, *P* = .028; Figure 2A). Figure 2B shows the OS in the two cohorts of patients according to HI‐E achievement. Since median survival was not reached in some groups, mean values are reported. No significant differences were observed according to rhEPO dose within the different subgroups (no responders: 75.1 months for SD and 67.4 months for HD, *P* = .63; responders: 78.1 months for SD and 91.4 months for HD, *P* = .27).

A multivariate analysis carried out adjusting for gender, age, rhEPO dose, transfusion dependency, ferritin, marrow blasts, WHO classification, and IPSS‐R score, OS was confirmed to be better for younger patients (HR = 0.62, 95% CI 0.52‐0.75, *P* < .001) for those with lower IPSS‐R score (HR = 0.64, 95% CI 0.51‐0.82, *P* < .001) and with transfusion independence (HR = 0.78, 95% CI 0.65‐0.94, *P* = .010) (Table S4).

### 3.5 Impact of clinical characteristics on response to HD or SD ESA doses

**Figure 3:**
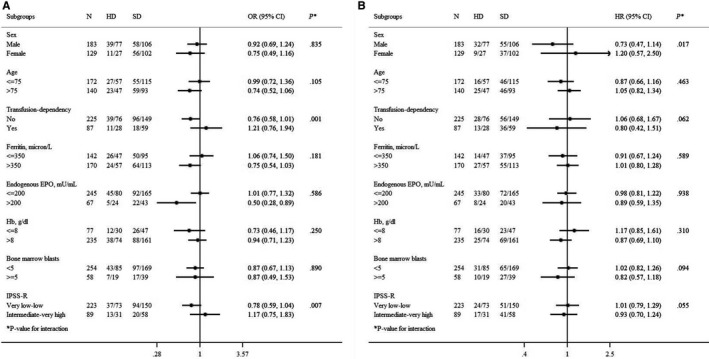
Forest plot of subgroup analyses of erythroid response (A) and overall survival (B) comparing rhEPO high dose (HD) vs rhEPO standard dose (SD) within the strata of each relevant clinical factor

Within the HD and SD cohorts, we also investigated whether a higher HI‐E rate or a longer OS could be associated with specific clinical characteristics. Figure 3A shows the subgroup analyses regarding HI‐E and Figure 3B the analysis regarding OS. Higher HI‐E rate was observed in HD cohort compared with SD cohort in both transfusion‐dependent patients and higher IPSS‐R risk categories (*P* = .001 and *P* = .007 respectively), but no significant impact on OS was detected in these subgroups of prognostically more severe patients.

### 3.6 Progression to AML according to ESA doses

Overall, 38 patients (12.2%) (14.4% in SD and 7.7% in HD group respectively), progressed to AML. Progression to AML was significantly higher among patients with transfusion dependency (19.5% vs 9.3% in nondependent patients, *P* = .02), with higher marrow blast count (27.6% for ≥5% vs 8.6% for <5, *P* < .001), without HI‐E (18.9% vs 6.1% for responders, *P* = .001), and with higher IPSS‐R risk categories (27.0% for intermediate‐very high‐risk and 6.3% for low‐very low‐risk scores, *P* = .001). Higher progression to AML, although not significant, was also observed among younger patients (15.1% ≤75 year vs 8.6% for >75 years, *P* = .085). A multivariate analysis adjusted for age, rhEPO dose, transfusion dependency, marrow blast percentage, WHO categories and IPSS‐R score, a higher blast percentage (>5%: OR = 1.91, 95% CI 1.01‐3.60, *P* = .047) and IPSS‐R score (intermediate or higher: OR = 1.85, 95% CI 1.16‐2.95, *P* = .010) were predictive of progression to AML. rhEPO doses were not associated with AML progression (HD: OR = 0.71, 95% CI 0.46‐1.10, *P* = .13).

When restricting the analysis to the 274 patients who did not progress to AML, multivariate analysis confirmed that rhEPO doses were not associated with HI‐E (OR = 0.90, 95% CI 0.69‐1.17, *P* = .44). Transfusion dependence was associated with the lack of response (no vs yes: OR = 1.54, 95% CI 1.15‐2.07, *P* = .004). Endogenous serum EPO levels (≤200 U/L vs >200 U/L: OR = 1.33, 95% CI 0.96‐1.85, *P* = .083) were weakly associated with HI‐E as well as IPSS‐R risk categories (very low‐low vs intermediate or higher: OR = 1.41, 95% CI 0.95‐2.09, *P* = .083).

### 3.7 Random intercept logistic regression analysis among propensity‐matched patients

The random intercept logistic regression modeling taking into account therapy and gender confirmed that rhEPO dose was not a predictive factor for response (HD vs SD: OR = 0.74, 95% CI 0.45‐1.22) (Table 3, model 1). No statistically significant association was also seen between rhEPO doses and progression to AML (HD vs SD: OR = 0.48, 95% CI 0.20‐1.11) (Table 3, model 3). Higher‐dose rhEPO treatment was not a positive prognostic factor for OS (HR = 0.82, 95% CI 0.55‐1.21) (Table 4, model 1). Similar results were obtained when the analysis was restricted to patients not progressing to AML, with an OR of 0.66 (95% CI 0.40‐1.10) for HI‐E (Table 3, model 2) and a HR of 0.92 (95% CI 0.59‐1.46) for OS (Table 4, model 2).

**Table 3:** Effect of treatment doses and gender on erythroid response rates and progression to acute myeloid leukemia estimated through the random intercept logistic regression modeling, after matching patients on propensity score derived from age, endogenous EPO, transfusion dependency, Hb, ferritin, and IPSS score

Variable	Model 1 (n = 312)		Model 2 (n = 274)		Model 3 (n = 312)	
	OR	95% CL (*P*‐value)	OR	95% CL (*P*‐value)	OR	95% CL (*P*‐value)
Treatment: higher vs standard doses	0.74	0.45‐1.22 (.235)	0.66	0.40‐1.10 (.114)	0.48	0.20‐1.11 (.086)
Gender: female vs male	0.92	0.57‐1.50 (.737)	0.89	0.54‐1.47 (.646)	0.89	0.43‐1.84 (.753)

Note

Model 1: erythroid response as outcome, all patients; Model 2: erythroid response as outcome, leukemia‐free patients; Model 3: leukemia as outcome.

Abbreviations: 95% CL: 95% confidence limits for OR; OR: odds ratio; *P*‐value: significance level of the likelihood ratio test.

**Table 4:** Effect of treatment doses and gender on overall survival estimated through the random intercept Cox regression modeling, after matching patients on propensity score derived from age, endogenous EPO, transfusion dependency, Hb, ferritin, and IPSS score

Variable	Model 1 (n = 312)		Model 2 (n = 274)	
	HR	95% CL (*P*‐value)	HR	95% CL (*P*‐value)
Treatment: higher vs standard doses	0.82	0.55‐1.21 (.318)	0.92	0.59‐1.46 (.747)
Gender: female vs male	0.51	0.34‐0.78 (.002)	0.50	0.31‐0.82 (.006)

Note

Model 1: all patients; Model 2: leukemia‐free patients.

Abbreviations: 95% CL, 95% confidence limits for HR; HR, hazard ratio; *P*‐value, significance level of the likelihood ratio test.

## 4 DISCUSSION

Our study shows that anemic MDS patients treated with different rhEPO doses (either SD or HD) achieve similar HI‐E rates, provided that they have similar clinical characteristics influencing the eligibility for treatment with ESAs.

These results apparently contrast with previous studies on MDS anemic patients indicating a higher response rate to ESAs, and in particular to rhEPO, when HD are employed^13^, ^14^ compared to SD.^10^ These latter poor results were mostly obtained in clinical studies performed in the early 90s using different but usually relatively low doses of rhEPO in the various subsets of MDS patients; in fact, in that early period trials actually enrolled a relevant proportion of patients with more advanced MDS, including subjects with RAEB and/or patients with a greater transfusional need. Most of these patients would be in present days classified as “high‐risk,” for whom to date treatment with ESAs is not generally considered an optimal choice.

Two meta‐analyses comparing studies performed in quite a long time lapse and with different dosing schedules in possibly heterogeneous groups of MDS patients indicated a possible superiority of HD of rhEPO vs SD. Data from 30 selected studies on MDS patients treated with ESAs at different dosing showed that HD of both rhEPO and darbepoetin induced higher HI‐E in lower‐risk MDS.^21^ However, in a subsequent meta‐analysis focused on rhEPO, among MDS patients treated with SD a significantly higher number of cases had marrow blasts >5% than cases treated with HD and SD patients treated had significantly higher endogenous serum EPO levels.^22^ Both these factors are clearly related to a poor response to rhEPO^9^ and possibly influenced the clinical outcome. Strictly selected MDS lower‐risk patients treated with SD had response rates equivalent to those observed with HD.^23^, ^24^ The recent randomized trial comparing safety and efficacy of rhEPO with placebo in low‐risk MDS patients used weight‐adjusted doses of rhEPO, substantially equivalent to SD.^25^

Erythroid response to ESAs is determined in fact by several predictive factors, and different clinical scores have been proposed during the years. Beyond the Nordic score,^17^ when only “low‐risk” MDS anemic patients with a blast count of less than 10% and with a low transfusion burden were treated with rhEPO,^26^ response rate was actually more than doubled comparing with previous studies.^10^ An IPSS‐R‐based predictive system has been more recently proposed considering also serum EPO and ferritin concentrations.^18^ It significantly predicted HI‐E after ESAs and it was recently already validated by a larger study.^27^ In the present study, we compared two cohorts of patients according to their eligibility to be treated with rhEPO applying a propensity score based on the clinical parameters influencing treatment choice. When the impact of different rhEPO doses was evaluated within such two homogenous cohorts the differences in response rates did not statistically differ. Transfusion‐dependent MDS patients and/or those with a higher IPSS‐R risk score in any case benefit more from HD than from SD, in terms of achievement of HI‐E. In these subsets of MDS patients, it would therefore be advisable to start treatment with HD ESAs, if other therapeutic options (ie, as example, hypomethylating agents) are not suitable or possible.

Our results are consistent with previous ones indicating longer OS in MDS patients who achieve HI‐E, at whichever rhEPO doses used.^8^, ^9^ We recently demonstrated that a trend for survival advantage is present for MDS patients with isolated erythroid dysplasia (RA/RARS/del5q) receiving ESAs for severe‐moderate anemia (8‐10 g/dL).^28^

Treatment with rhEPO—irrespectively of the doses used—do not have significant impact on progression to AML, which was higher among MDS patients not responding to rhEPO, transfusion‐dependent patients and with higher IPSS‐R risk scores, confirming previous results.^29^, ^30^

In conclusion, although with the limits of a retrospective analysis and a relatively limited number of cases, our study indicates that the SD of rhEPO is as effective as HD in improving anemia in MDS patients stratified according to the propensity score of treatment, with the exception of transfusion‐dependent patients and with higher IPSS‐R risk scores. Moreover, different doses have the same effects on OS and risk of AML transformation. Our observations may lead to a wiser use of SD of rhEPO, reserving HD to the above indicated subcategories of MDS patients, limiting the economic impact of the treatment. It has been shown that early use of ESAs can significantly delay the onset of a transfusion need in lower‐risk MDS patients, and is associated with a reduced chance of death in responding patients.^31^ This further supports the relevance of our analysis in view of increased appropriateness in early therapy with ESAs.

## CONFLICT OF INTEREST

EB served as a member of local advisory board for Janssen‐Cilag, Novartis, and Celgene. EA has received honoraria from Novartis and Celgene, involvement in local advisory boards for Jazz Pharmaceuticals and Roche and participation in DMC for Celgene and Vertex Pharmaceuticals Incorporated and CRISPR Therapeutics. DC has received honoraria from Novartis and Celgene. CF has received research funding, advisory committees, and speaker fees from Novartis, Janssen, and Celgene. PM received honoraria from and participation to advisory boards for Janssen e Amgen. ENO received research funding from Janssen‐Cilag. FP served as a member of advisory board for Novartis. VS has received honoraria from Celgene, Janssen, and Novartis. Advisory boards for Celgene, Janssen, Abbvie, Astex, Karyopharma, Acceleron. RAF, CS, BA, MB, TC, MC, MC, EC, ADC, PD, DF, DG, RML, EM, EM, MM, AP, FS, AS, MS, and RT have nothing to disclose.

## AUTHOR CONTRIBUTION

EB and VS designed research, analyzed data, and wrote the paper. RAF and MB analyzed data and performed statistical analysis. CS, DG, and EM collected data. BA, EA, TC, MC, MC, DC, MC, EC, ADC, PD, FP, DF, CF, RML, EM, MM, PM, ENO, AP, FS, AS, MS, and RT contributed with the patient clinical data. All authors gave their final approval of manuscript.
