# Supplementary material for: Effects of different doses of erythropoietin in patients with myelodysplastic syndromes: A propensity score‐matched analysis
Source: Cancer Med. 2019 Oct 27;8(18):7567–76. doi: 10.1002/cam4.2638 (PMC6912022; doi:10.1002/cam4.2638)
Supplement: Supplementary file 1 [file CAM4-8-7567-s001.docx]

**Supporting information**

Supporting Table 1. response to therapy according to clinical characteristics of study population

(matched patients n. 312)

|  |  | | p |
| --- | --- | --- | --- |
|  | No response | Response |  |
|  | N (%) | N (%) |  |
| *Total* | 148 (47.4) | 164 (52.6) |  |
| *Sex* |  |  | 0.91 |
| male | 86 (47.0) | 97 (53.0) |  |
| female | 62 (48.1) | 67 (51.9) |  |
| *Age* |  |  | 0.068 |
| <=75 | 90 (52.3) | 82 (47.7) |  |
| >75 | 58 (41.4) | 82 (58.6) |  |
| *WHO classification* |  |  | 0.042 |
| RA | 44 (41.9) | 61 (58.1) |  |
| RARS | 17 (39.5) | 26 (60.5) |  |
| RCMD | 42 (45.7) | 50 (54.3) |  |
| RAEB1 | 22 (52.4) | 20 (47.6) |  |
| RAEB2 | 6 (75.0) | 2 (25.0) |  |
| MDS with isolated 5q- | 12 (80.0) | 3 (20.0) |  |
| MDS-U | 4 (66.7) | 2 (33.3) |  |
| *Hemoglobin (g/dL)* |  |  | 0.60 |
| ≤8 | 39 (50.6) | 38 (49.4) |  |
| >8 | 109 (46.4) | 125 (53.6) |  |
|  |  |  | 1.0 |
| <=10 | 135 (47.4) | 150 (52.6) |  |
| >10 | 13 (48.1) | 14 (51.9) |  |
| *Bone marrow blasts(%)* |  |  | 0.08 |
| *<5* | 114 (44.9) | 140 (55.1) |  |
| *≥5* | 34 (58.6) | 24 (41.4) |  |
| *Transfusion-dependency* |  |  | <0.001 |
| No | 90 (40.0) | 135 (60.0) |  |
| Yes | 58 (66.7) | 29 (33.3) |  |
| *IPSS-R score risk* |  |  | <0.001 |
| Very low-low | 92 (41.3) | 131 (58.7) |  |
| Intermediate- very high | 56 (62.9) | 33 (37.1) |  |
| *Ferritin (μg/L)* |  |  | 0.82 |
| ≤350 | 66 (46.5) | 76 (53.5) |  |
| >350 | 82 (48.2) | 88 (51.8) |  |
| *EPO levels ( mU/mL)* |  |  | 0.027 |
| ≤200 | 108 (44.1) | 137 (55.9) |  |
| >200 | 40 (59.7) | 27 (40.3) |  |
| *rhEPO Therapy* |  |  | 0.28 |
| Standard doses | 94 (45.2) | 114 (54.8) |  |
| Higher doses | 54 (51.9) | 50 (48.1) |  |

Supporting Table 2- Multivariate analysis for erythroid response to therapy

|  | Odds ratio  (OR) | 95%CI | p |
| --- | --- | --- | --- |
| *WHO classification* |  |  | 0.26 |
| RA | 1.0 |  |  |
| RARS | 1.33 | 0.62-2.83 | 0.46 |
| RCMD | 1.39 | 0.75-2.56 | 0.29 |
| RAEB1 | 1.88 | 0.77-4.61 | 0.17 |
| RAEB2 | 0.86 | 0.17-4.47 | 0.86 |
| MDS with isolated 5q- | 0.23 | 0.07-0.79 | 0.020 |
| MDS-U | 0.96 | 0.19-4.75 | 0.96 |
| *Bone marrow blasts(%)* |  |  | 0.82 |
| *>=5* | 1.0 |  |  |
| *<5* | 1.06 | 0.62-1.83 |  |
| *Transfusion-dependency* |  |  | <0.001 |
| Yes | 1.0 |  |  |
| No | 1.71 | 1.30-2.25 |  |
| *IPSS-R score risk* |  |  | 0.035 |
| Intermediate- very high | 1.0 |  |  |
| Very low-low | 1.45 | 1.03-2.06 |  |
| *EPO levels ( mU/mL)* |  |  | 0.23 |
| >200 | 1.0 |  |  |
| <=200 | 1.20 | 0.89-1.63 |  |
| *rhEPO Therapy* |  |  | 0.39 |
| Standard doses | 1.0 |  |  |
| Higher doses | 0.86 | 0.67-1.11 |  |

Supporting Table 3. Overall survival according to clinical characteristics of study population ( matched patients n. 312)

|  |  | |  |
| --- | --- | --- | --- |
|  | Median (mo) | 95%CI | p |
| *Total* | 64.6 | 49.2-79.9 |  |
| *Sex* |  |  | 0.010 |
| male | 46.8 | 32.0-61.6 |  |
| female | 86.2 | 56.0-116.4 |  |
| *Age* |  |  | <0.001 |
| <=75 | 88.0 | 86.2-108.3 |  |
| >75 | 36.4 | 27.4-45.4 |  |
| *WHO classification* |  |  | 0.002 |
| RA | 83.1 | 61.0-105.1 |  |
| RARS | 95.1 | 76.2-114.0 |  |
| RCMD | 39.7 | 25.9-53.5 |  |
| RAEB1+RAEB2 | 31.0 | 16.2-45.9 |  |
| MDS with isolated 5q- | 57.5 | 37.0-78.1 |  |
| *Hemoglobin (g/dL)* |  |  | 0.14 |
| ≤8 | 42.1 | 14.6-69.5 |  |
| >8 | 66.5 | 50.5-82.4 |  |
|  |  |  | 0.16 |
| <=10 | 61.7 | 46.5-77.0 |  |
| >10 | 114.6 | 98.6-120.4 |  |
| *Bone marrow blasts(%)* |  |  | <0.001 |
| *<5* | 74.5 | 59.4-89.6 |  |
| *≥5* | 31.2 | 23.1-39.2 |  |
| *Transfusion-dependency* |  |  | <0.001 |
| No | 80.2 | 64.0-96.4 |  |
| Yes | 32.5 | 24.6-40.3 |  |
| *IPSS-R score risk* |  |  | p<0.001 |
| Very low-low | 80.2 | 78.9-97.0 |  |
| Intermediate- very high | 35.2 | 26.0-44.4 |  |
| *Ferritin (μg/L)* |  |  | 0.010 |
| ≤350 | 86.2 | 59.4-113.0 |  |
| >350 | 45.6 | 30.0-61.4 |  |
| *EPO levels ( mU/mL)* |  |  | 0.73 |
| ≤200 | 58.2 | 40.9-75.4 |  |
| >200 | 74.5 | 48.6-100.3 |  |
| *Response to therapy* |  |  | 0.028 |
| No | 52.3 | 40.1-64.5 |  |
| Yes | 86.2 | 65.7-106.7 |  |
| *rhEPO Therapy* |  |  | 0.78 |
| Standard doses | 58.5 | 42.3-74.7 |  |
| High doses | 80.2 | 31.1-129.3 |  |

Supporting Table 4. Multivariate analysis for overall survival

|  | Hazard ratio  (HR) | 95%CI | p |
| --- | --- | --- | --- |
| *Sex* |  |  | 0.037 |
| Male | 1.0 |  |  |
| Female | 0.66 | 0.45-0.97 |  |
| *Age* |  |  | <0.001 |
| >75 | 1.0 |  |  |
| <=75 | 0.62 | 0.52-0.75 |  |
| *WHO classification* |  |  | 0.46 |
| RA | 1.0 |  |  |
| RARS | 0.87 | 0.62-1.22 |  |
| RCMD | 0.71 | 0.43-1.16 |  |
| RAEB1& RAEB2 | 1.14 | 0.81-1.60 |  |
| MDS with isolated 5q- | 1.35 | 0.78-2.35 |  |
| *Bone marrow blasts(%)* |  |  | 0.78 |
| *>=5* | 1.0 |  |  |
| *<5* | 1.05 | 0.75-1.47 |  |
| *Transfusion-dependency* |  |  | 0.010 |
| Yes | 1.0 |  |  |
| No | 0.78 | 0.65-0.94 |  |
| *IPSS-R score risk* |  |  | <0.001 |
| Intermediate- very high | 1.0 |  |  |
| Very low-low | 0.64 | 0.51-0.82 |  |
| *Ferritin (μg/L)* |  |  | 0.013 |
| >350 | 1.0 |  |  |
| <=350 | 0.79 | 0.66-0.95 |  |
| *rhEPO Therapy* |  |  | 0.34 |
| Standard doses | 1.0 |  |  |
| High doses | 0.91 | 0.75-1.10 |  |
